# Variant surface glycoproteins from Venezuelan trypanosome isolates are recognized by sera from animals infected with either *Trypanosoma evansi* or *Trypanosoma vivax*

**DOI:** 10.1016/j.vetpar.2014.11.004

**Published:** 2015-01-15

**Authors:** Rocío Camargo, Adriana Izquier, Graciela L. Uzcanga, Trina Perrone, Alvaro Acosta-Serrano, Liomary Carrasquel, Laura P. Arias, José L. Escalona, Vanessa Cardozo, José Bubis

**Affiliations:** a Fundación Instituto de Estudios Avanzados IDEA, Caracas, Venezuela; b Universidad Simón Bolívar, Departamento de Biología Celular, Caracas, Venezuela; c Universidad Central de Venezuela, Instituto de Ciencia y Tecnología de Alimentos, Caracas, Venezuela; d Instituto Venezolano de Investigaciones Científicas IVIC, Centro de Biofísica y Bioquímica, Caracas, Venezuela; e Parasitology Department, Liverpool School of Tropical Medicine, Liverpool, UK; f Universidad Simón Bolívar, Departamento de Química, Caracas, Venezuela

**Keywords:** ABTS, 2,2′-azino-bis(3-ethylbenzothiazoline-6-sulphonic acid), BCIP, 5-bromo-4-chloro-3 indolyl phosphate, Con A, concanavalin A, CRD, cross-reacting determinant, DAB, diaminobenzidine, IEF, isoelectric focusing, LC/ESI-MS/MS, liquid chromatography/electrospray ionization-tandem mass spectrometry, NBT, nitro blue tetrazolium, o-PDM, N-N′-1,2 phenylenedimaleimide, p-PDM, N-N′-1,4 phenyllenedimaleimide, PMSF, phenyl methyl sulfonyl fluoride, RAPD, random amplification polymorphic DNA, Rf, relative mobility, RoTat, Rode Trypanozoon antigen type, SDS-PAGE, sodium dodecyl sulfate-polyacrylamide gel electrophoresis, sulfo-SMCC, sulfosuccinimidyl 4-(N-maleimidomethyl)cyclohexane-1-carboxylate, VSG, variant surface glycoproteins, Animal trypanosomosis, *Trypanosoma evansi*, *Trypanosoma vivax*, Variant surface glycoproteins, Diagnosis, Cross-reacting antigens

## Abstract

Salivarian trypanosomes sequentially express only one variant surface glycoprotein (VSG) on their cell surface from a large repertoire of *VSG* genes. Seven cryopreserved animal trypanosome isolates known as TeAp-ElFrio01, TEVA1 (or TeAp-N/D1), TeGu-N/D1, TeAp-Mantecal01, TeGu-TerecayTrino, TeGu-Terecay03 and TeGu-Terecay323, which had been isolated from different hosts identified in several geographical areas of Venezuela were expanded using adult albino rats. Soluble forms of predominant VSGs expressed during the early infection stages were purified and corresponded to concanavalin A-binding proteins with molecular masses of 48–67 kDa by sodium dodecyl sulfate-polyacrylamide gel electropohoresis, and p*I* values between 6.1 and 7.5. The biochemical characterization of all purified soluble VSGs revealed that they were dimers in their native form and represented different gene products. Sequencing of some of these proteins yielded peptides homologous to VSGs from *Trypanosoma* (Trypanozoon) *brucei* and *Trypanosoma* (Trypanozoon) *evansi* and established that they most likely are mosaics generated by homologous recombination. Western blot analysis showed that all purified VSGs were cross-reacting antigens that were recognized by sera from animals infected with either *T*. *evansi* or *Trypanosoma* (Dutonella) *vivax*. The VSG glycosyl-phosphatidylinositol cross-reacting determinant epitope was only partially responsible for the cross-reactivity of the purified proteins, and antibodies appeared to recognize cross-reacting conformational epitopes from the various soluble VSGs. ELISA experiments were performed using infected bovine sera collected from cattle in a Venezuelan trypanosome-endemic area. In particular, soluble VSGs from two trypanosome isolates, TeGu-N/D1 and TeGu-TeracayTrino, were recognized by 93.38% and 73.55% of naturally *T. vivax*-infected bovine sera, respectively. However, approximately 70% of the sera samples did not recognize all seven purified proteins. Hence, the use of a combination of various VSGs for the diagnosis of animal trypanosomosis is recommended.

## Highlights

• Soluble forms of VSGs from seven Venezuelan animal trypanosomes were purified and characterized.
• All purified soluble VSGs exhibited cross-reactivity with *Trypanosoma vivax*.
• Anti-VSG antibodies behaved as markers of infection for non-tsetse transmitted trypanosomes.
• All purified soluble VSGs can be used as diagnostic reagents for bovine trypanosomosis.

## 1 Introduction

Salivarian trypanosomes have a dense surface coat composed predominantly of a unique type of variant surface glycoproteins (VSG), which shields the invariant surface proteins from host immune system effectors and prevents complement activation (Turner et al., 1985). Despite the high number of *VSG* and pseudo-*VSG* genes within the trypanosome genome, each trypanosome expresses only one *VSG* gene at a time from a telomeric expression site, while the remaining *VSG* genes are transcriptionally silent. VSG itself is highly immunogenic and elicits specific trypanocidal responses from the host's immune system. Trypanosomes escape this immune response by switching their dense protective VSG coat. As the infection progresses, a high host antibody titer to a particular VSG causes clearance of trypanosomes expressing that particular VSG. However, trypanosomes that switch VSG expand as a new population until they in turn are recognized by the host immune system. Consequently, trypanosomes persist in their mammal hosts due to their antigenic variation strategy (Barry and McCulloch, 2001, Taylor and Rudenko, 2006, Jackson et al., 2012).

In Venezuela, two non-tsetse transmitted salivarian trypanosome species, *Trypanosoma* (Trypanozoon) *evansi* and *Trypanosoma* (Dutonella) *vivax*, are the main cause of animal trypanosomosis in livestock and generate a significant economic impact. In Venezuela, both *T. e*v*ansi* and *T. vivax* are mechanically transmitted by biting insects including *Tabanus*, *Cryptotylus*, and *Stomoxys* species. In Africa, *T. vivax* is highly prevalent in both tsetse-infested and tsetse-free regions. The cyclical transmission of *T. vivax* is limited to tsetse flies; mechanical transmission by other biting flies allows *T. vivax* to spread in some tsetse-free African regions where it is disseminated by tabanids and stomoxes. Information available for the Llanos of Venezuela indicates that ∼7% of horses suffer active infection with *T. evansi* (García et al., 2000, Castellanos et al., 2010, Forlano et al., 2011). It has been calculated that losses owing to horse mortality caused by this hemoparasitosis would have amounted to US$ 7,486,000 for this region in 2008 (Moreno et al., 2013). García et al. (2005) have shown by bloodsmear examinations, microhaematocrit centrifugations and immunological assays that 6.7%, 11.4% and 39.5% of Venezuelan blood samples from water buffaloes and other livestock contained trypanosomes. Moreover, their results indicated that about 20% of the blood samples contained *T. vivax* (García et al., 2005). García et al. (2006) also evaluated the seroprevalence of trypanosomosis and the prevalence of current trypanosome infection in water buffaloes from the most important livestock areas of Venezuela. Of the 644 animals investigated, 6.2% were found infected with trypanosomes by blood centrifugation, and 30.4% were found positive for anti-trypanosome antibodies. The results of the PCR-based assay indicated that 92.5% of the animals with current infections were infected with *T*. *vivax* (García et al., 2006). In addition, these diagnostic studies demonstrated that the infection caused by *T. vivax* was practically asymptomatic in Venezuelan endemic areas (García et al., 2005, García et al., 2006). Greif et al. (2013) conducted a RNA-seq analysis of the Venezuelan *T. vivax* LIEM-176 isolate. This study described proteins that were differentially expressed between the LIEM-176 isolate and the reference *T. vivax* Zaria Y486 Nigerian isolate (Greif et al., 2013). Recently, García et al. (2014) investigated *T. vivax* genetic diversity, population structure and the source of outbreaks through the microsatellite multiloci genotype analysis of isolates from across South America and West Africa. Their results supported clonal propagation, and were consistent with the hypothesis that the *T. vivax* isolates from South America derived from common ancestors recently introduced from West Africa (García et al., 2014). Although *Trypanosoma* (Trypanozoon) *equiperdum* has not been reported in Venezuela, Perrone et al. (2009) have proposed that two Venezuelan trypanosome isolates from horses, TEVA1 (also known as TeAp-N/D1) and TeGu-N/D1, previously thought to be *T. evansi*, could belong to a morphologically indistinguishable species within the subgenus Trypanozoon, such as *T. equiperdum*.

Various reports have shown a very high immunological cross-reactivity between trypanosome species, in particular *T. evansi* and *T. vivax* (Desquesnes and Tresse, 1996, Aray et al., 1998). Although in vivo outbred murine models of trypanosomosis (CD-1, RjOrl:Swiss mice) have been developed using the IL 1392 strain of *T. vivax* that was originally derived from the Y486 isolate from Africa (Leeflang et al., 1976, Chamond et al., 2010, Blom-Potar et al., 2010), and in vitro non-infective *T. vivax* epimastigote axenic cultures have been reported using the same IL 1392 strain (D’Archivio et al., 2011), the production of *T. vivax* antigens continues to be a limiting factor because most *T. vivax* stocks are restricted to large animals such as cattle, sheep, goats, horses, donkeys and pigs, and possess relatively low level parasitaemias. In contrast, rodents can be readily infected in the laboratory with any stock of *T. evansi* or *T. equiperdum* to obtain high quantities of parasites to prepare antigens for serological tests. For that reason, we have focused on the diagnosis of *T. vivax*-caused animal trypanosomosis by using cross-reacting antigens isolated from other trypanosomes (Uzcanga et al., 2002, Uzcanga et al., 2004, Camargo et al., 2004, Velásquez et al., 2014). Interestingly, a 64-kDa glycosylated cross-reacting antigen from the TEVA1 isolate was proven to represent the soluble form of a VSG (Uzcanga et al., 2004). This result was consistent with several studies that have demonstrated that entire VSG molecules, VSG peptides and VSG mimotopes can be used to diagnose *Trypanosoma* (Trypanozoon) *brucei* and *T. evansi* infections (Bajyana Songa and Hamers, 1988, Penchenier et al., 2003, Ngaira et al., 2004, Sengupta et al., 2012, Van Nieuwenhove et al., 2012, Van Nieuwenhove et al., 2013). In this study, we investigated the potential use of VSG variants as diagnostic reagents for the detection of trypanosomosis caused by *T. vivax* and *T. evansi*, and examined whether the soluble form of these VSG antigens contained common epitopes recognized by sera from animals infected with either of these species of trypanosomes.

## 2 Materials and methods

### 2.1 Materials

Reagents were purchased from the following sources: middle range molecular weight protein markers, anti-mouse IgG horseradish peroxidase conjugate, 5-bromo-4-chloro-3 indolyl phosphate (BCIP), nitro blue tetrazolium (NBT), *Promega*; anti-rabbit IgG alkaline phosphatase conjugate, anti-bovine IgG horseradish peroxidase conjugate, anti-bovine IgG alkaline phosphatase conjugate, anti-equine IgG alkaline phosphatase conjugate, anti-mouse IgG alkaline phosphatase conjugate, diaminobenzidine (DAB), horseradish peroxidase type VI-A, fibrous DEAE-cellulose, benzamidine, iodoacetamide, phenyl methyl sulfonyl fluoride (PMSF), N-N′-1,2 phenylenedimaleimide (o-PDM), N-N′-1,4 phenyllenedimaleimide (p-PDM), gel filtration molecular weight protein marker kit, *Staphylococcus aureus* V8 protease, concanavalin A (Con A), 2,2′-azino-bis(3-ethylbenzothiazoline-6-sulphonic acid) (ABTS), methyl-α-d-mannopyranoside, methyl-α-d-glucopyranoside, *Sigma*; Q-Sepharose, S-Sepharose, Sefacryl S-300, *Pharmacia*; pre-stained high molecular weight protein markers, *Gibco BRL*; sulfosuccinimidyl 4-(N-maleimidomethyl)cyclohexane-1-carboxylate (sulfo-SMCC), nitrocellulose (0.45 μm pore size), *Pierce;* broad range isoelectric focusing calibration kit (3–10), Immobilin dry strips (pH 5–8), ampholites (pH 3–10), *BioRad*. All other chemicals were of the highest quality grade available.

### 2.2 Parasites

Seven cryopreserved animal trypanosome isolates were used that had been isolated from different hosts from different geographical areas of Venezuela. The details about each parasite isolate are presented in Table 1. Two horse isolates, TEVA1 and TeGu-N/D1, formerly considered to be *T. evansi*, showed distinctive random amplification polymorphic DNA (RAPD) patterns and may belong to the *T. equiperdum* species (Perrone et al., 2009). All the remaining five isolates clearly belong to the *T. evansi* species (Perrone et al., 2009).

**Table 1:** Summary information of the animal trypanosome isolates used in this study.^a^

Sample no.	Isolate name	Natural host	Geographical location (Ranch, Municipality, State)	Year of isolation
1	TeAp-ElFrio01	Capybara (*Hydrochoerus hydrochaeris*)	El Frio Ranch, Muñoz Municipality, Apure State	April 1992
2	TEVA1 or TeAp-N/D1^b^	Horse (*Equus ferus caballus*)	N/D^d^, Apure State	N/D^d^
3	TeGu-N/D1^c^	Horse (*Equus ferus caballus*)	N/D^d^, Guárico State	N/D^d^
4	TeAp-Mantecal01	Horse (*Equus ferus caballus*)	Mantecal Ranch, Muñoz Municipality, Apure State	December 1996
5	TeGu-TerecayTrino	Donkey (*Equus africanus asinus*)	Terecay Ranch, Camaguán Municipality, Guárico State	November 1999
6	TeGu-Terecay03	Donkey (*Equus africanus asinus*)	Terecay Ranch, Camaguán Municipality, Guárico State.	November 1999
7	TeGu-Terecay323	Donkey (*Equus africanus asinus*)	Terecay Ranch, Camaguán Municipality, Guárico State	November 1999

a These isolates were previously characterized by Perrone et al. (2009).

b Donated by the Center of Biomedical and Veterinary Studies of the Simón Rodríguez National Experimental University, Caracas, Miranda State.

c Donated by the Faculty of Veterinary Sciences of the Central University of Venezuela, Maracay, Aragua State.

d N/D, not determined.

All isolates were passaged using adult albino rats (Sprague-Dawley), which were infected by intraperitoneal inoculation with 0.30 ml of infected blood, containing approximately 10^8^ trypanosomes. When the parasitaemia reached approximately 10^7^–10^9^ trypanosomes/ml, blood was extracted by cardiac puncture using 0.5 M EDTA as an anticoagulant. Parasites were separated by anion-exchange chromatography using a fibrous DEAE-cellulose column, as previously described (Lanham and Godfrey, 1970). Parasites were washed and purified according to Camargo et al. (2004).

### 2.3 Animal sera

Sera were obtained from 16 naturally infected, parasite positive animals (11 cows, two donkeys, two capybaras and one buffalo) from El Frio ranch, Muñoz Municipality, Apure State. Sera from six confirmed non-infected animals living in Venezuelan trypanosomosis-free areas were used as negative controls (three cows, one donkey and one capybara from local farms; and one racehorse from La Rinconada Racetrack, Caracas). Sera from two animals experimentally infected with trypanosomes were used as positive controls; one horse was infected with *T. evansi* TeAp-El Frio01 and one cow was infected with *T. vivax* LIEM-176. Naturally and experimentally infected animals were examined for trypanosome infection using the micro-haematocrit technique (Woo, 1970). Animals were also diagnosed as negative or positive for trypanosomosis by indirect ELISA (Aray et al., 1998), using the clarified antigenic fraction from the TEVA1 isolate as the antigen source (Uzcanga et al., 2002). For the diagnosis of bovine trypanosomosis, sera from 121 cows were obtained from La Candelaria, La Esperanza, El Paradero and La Loma ranches, José Tadeo Monagas Municipality, Guárico State, which is a trypanosomosis-endemic area in Venezuela. All animal sera were stored at −20 °C.

### 2.4 Purification of the soluble form of VSG from animal trypanosomes

Parasites were homogenized on ice by sonication (four cycles of 30 s each, with a 30 s resting period between) using 10 ml of 5 mM Tris–HCl pH 7.6, 1 mM benzamidine 1 mM iodoacetamide, 1 mM PMSF and 5 mM EDTA. The homogenate was incubated for 5 min at room temperature and then centrifuged at 100,000 × *g*, for 30 min at 4 °C to obtain the supernatant and pellet fractions. The pellet fraction was extracted, incubated for 5 min at room temperature, and centrifuged as described above. Both resulting supernatants were collected and loaded onto a Q-Sepharose column (40 ml) connected in tandem with a S-Sepharose column (15 ml), according to the procedure described previously (Uzcanga et al., 2002, Uzcanga et al., 2004). Under these conditions, the soluble VSG was eluted in the non-adhering fraction. The eluted proteins were monitored at 280 nm and the fractions were stored at −20 °C until use.

### 2.5 Detection of concanavalin A-binding glycoproteins

The protocol of Wood and Sarinama (1975) was used to detect glycoproteins. In brief, the nitrocellulose membranes with the transferred proteins were initially placed in a TBST solution [50 mM Tris–HCl (pH 8.0), 150 mM NaCl, 0.1% (v/v) Tween 20] containing 1% (w/v) gelatin for 1 h. Following incubation for 45 min with 0.5 mg/ml Con A in TBST, the nitrocellulose sheets were incubated for 45 min with horseradish peroxidase (0.1 mg/ml in TBST). The reactions were developed using DAB and hydrogen peroxide and stopped using distilled water. Parallel experiments in which the nitrocellulose membranes were treated with Con A, previously incubated with 0.2 M or 0.5 M methyl-α-d-mannopyranoside, methyl-α-d-glucopyranoside, or a mixture of both carbohydrates, were included as controls.

### 2.6 Sequencing of VSG polypeptides by liquid chromatography/electrospray ionization-tandem mass spectrometry (LC/ESI-MS/MS)

Samples of purified soluble forms of VSG (∼10–20 μg) were digested overnight with trypsin. The resulting peptides were analyzed by LC/ESI-MS/MS using the Ultimate 3000 (Dionex Inc.) nanoLC system coupled to a 4000 QTRAP (Applied Biosystems) (McMahon et al., 2010), at the University of Dundee Fingerprints Proteomics Facility. MASCOT was employed for the identification of the tryptic peptides, after searching the NCBInr and the *T. brucei brucei* genome database as there is not yet a complete *T. evansi* genomic database available.

### 2.7 Gel filtration chromatography

The purified proteins were applied to a Sephacryl S-300 size-exclusion column [Total volume (*V*_*t*_) = 188 ml] previously equilibrated with 50 mM Tris–HCl (pH 8.0), 150 mM NaCl, 5 mM β-mercaptoethanol, employing a flow rate of 0.175 ml/min. The column was calibrated using protein standards with molecular masses ranging from 12.4 to 200 kDa. The excluded (*V*_0_) and included volumes were determined by chromatographing blue dextran and potassium dichromate, respectively. The eluting fractions were separated by sodium dodecyl sulfate-polyacrylamide gel electrophoresis (SDS-PAGE). The elution volume (*V*_*e*_) was measured for each protein, and the corresponding distribution coefficient was calculated using the following equation:

$$K_{av}=\frac{V_{e}-V_{0}}{V_{t}-V_{0}}$$

The molecular weight of each purified VSG was empirically determined by plotting the *K*_*av*_ value of each standard versus the logarithm of its molecular weight.

### 2.8 Cross-linking of the soluble VSG forms purified from different trypanosome isolates

Samples of the purified soluble form of each VSG (8 μg) were incubated with different cross-linking reagents, namely sulfo-SMCC (5 mM), *o*-PDM (5 mM) or *p*-PDM (5 mM), for 1 h, at room temperature. Sulfo-SMCC stock solution was freshly prepared in water, while *o*-PDM and *p*-PDM stock solutions were freshly prepared in dimethyl sulfoxide. The reaction with sulfo-SMCC was carried out in 10 mM sodium phosphate (pH 7.2), whereas the reactions with both phenylenedimaleimides were performed in 25 mM Tris–HCl (pH 7.5) and 2.5 mM magnesium acetate. For controls, samples of the soluble form of each VSG were incubated with only the corresponding buffers, in the presence or absence of the appropriate amount of dimethyl sulfoxide. All samples were separated by SDS-PAGE and the cross-linked products were analyzed by silver staining.

### 2.9 2D isoelectric focusing/SDS-PAGE analysis of purified VSGs

Samples of the soluble form of each purified VSG (10 μg) were precipitated with cold 80% (v/v) acetone and 0.07% (v/v) β-mercaptoethanol and then resuspended in a solution containing 0.5% (v/v) ampholytes (pH 3–10), 8 M urea, 0.5% (v/v) Triton X-100 and 1.3 mM dithiothreitol. Isoelectric focusing (IEF) was performed in a Protean IEF Cell (Bio-Rad) following the instructions of the supplier, using polyacrylamide IPG 7 cm strips (ReadyStrip™, Bio-Rad) containing a linear ampholyte gradient (pH 5–8). Following the initial fractionation, the strips were incubated with 350 mM Tris–HCl (pH 6.8), 6 M urea, 30% (v/v) glycerol, 1.7% (w/v) SDS and 1% (w/v) dithiothreitol for 10 min at room temperature, and then with 350 mM Tris–HCl (pH 6.8), 6 M urea, 30% (v/v) glycerol, 1.7% (w/v) SDS and 2.5% (w/v) iodoacetamide, for 10 min at room temperature. The strips were placed on top of a 1 mm thick slab gel containing 10% (w/v) polyacrylamide and the second dimension was performed as described by Laemmli (1970). After silver staining the gel, the isoelectric points of the isoforms of the purified proteins were empirically determined by plotting the p*I* value of each of the 2D standard protein markers versus their relative mobility (*Rf*).

### 2.10 Immunoblotting

For Western blot analyses, proteins were transferred from the gels to nitrocellulose sheets as described previously (Towbin et al., 1979). The nitrocellulose filters were incubated with animal sera (dilution 1:100) or polyclonal antibodies, which were prepared in mice ascitic fluid (Overkamp et al., 1988, Cartledge et al., 1992) (dilution 1:5000) or in rabbit serum (dilution 1:400) against the soluble VSG form of the TEVA1 isolate. The membranes were then treated with the appropriate dilution of alkaline phosphatase-conjugated secondary antibodies against equine, bovine, mouse or rabbit IgG. On the basis of evolutionary relationships, secondary antibodies against equine, bovine, and mouse IgG were used when sera from donkeys, buffalo, and capybaras, respectively, were employed as the primary antibodies. Polypeptide bands were visualized by the addition of NBT and BCIP, according to the manufacturer's instructions.

### 2.11 Mild acid treatment

To assess the contribution of the glycosyl-phosphatidylinositol cross-reacting determinant (CRD) epitope in the sera cross-reactivity against the VSG samples, the purified soluble form of each VSG (50 μg) was treated for 1 h at room temperature with 1 M HCl as described previously (Schneider et al., 1990). Untreated samples were included as controls. After neutralization with 1 M NaOH, samples were reduced by the addition of 1% (v/v) β-mercaptoethanol and heated at 100 °C for 5 min. Samples were separated by SDS-PAGE and transferred to nitrocellulose sheets. The sheets were cut into 4 mm strips and were individually developed to evaluate the recognition of the soluble VSG forms by sera from animals naturally or experimentally infected with trypanosomes. Membranes were also developed using rabbit polyclonal antibodies (dilution 1:400) against the soluble VSG forms of the TEVA1 isolate. Western blotting was performed as described above.

### 2.12 Partial digestions of the soluble VSG form of each trypanosome isolate with *S. aureus* V8 protease

The purified soluble VSG forms of the seven trypanosome isolates were partially digested with *S. aureus* V8 protease in 50 mM Tris–HCl (pH 8.1), following the procedure described by Down et al. (1991). Samples containing 40 μg of each protein were precipitated with 80% (v/v) cold acetone (1 ml, at −20 °C for 10 min). The reactions were centrifuged at 14,000 × *g* for 5 min, and the supernatants were removed. The precipitates were reconstituted with 50 mM Tris–HCl (pH 8.1). Two different concentrations of the protease were employed: (a) 1:25 protease:protein (1.12 units) and (b) 1:75 protease:protein (0.37 units). The samples were incubated for 4.5 h at room temperature. Reactions were terminated by heating at 100 °C for 5 min in Laemmli sample buffer (1970). The resulting proteolytic products were separated by SDS-PAGE on a 15% (w/v) polyacrylamide gel. Sample aliquots were transferred to nitrocellulose sheets for subsequent Western blot analysis.

### 2.13 Diagnosis of bovine trypanosomosis by indirect ELISA using the purified soluble VSG forms as cross-reacting antigens

Indirect ELISA was undertaken according to the method described by Aray et al. (1998). ELISA plates were sensitized with the purified proteins (1 μg of protein/well), diluted in carbonate-bicarbonate buffer (pH 9.6), and left overnight at 4 °C in a humid chamber. Blocking buffer (PBS containing 0.1% (v/v) Tween 20 and 2% (w/v) gelatin) was applied in excess to each well for 1 h, at 37 °C. Bovine sera (121 samples) obtained from an endemic area in the Guárico State, Venezuela, were diluted 1:100 in blocking buffer. A 100 μl aliquot of the diluted serum was added per well. After an extensive wash, 100 μl of horseradish peroxidase-conjugated secondary antibody against bovine IgG (dilution 1:2000 in blocking buffer) was added to each well. The colorimetric reaction was developed after adding 100 μl of a solution containing 10% (w/v) ABTS and 0.0075% (v/v) hydrogen peroxide in 0.05 M phosphate-citrate buffer (pH 5).

### 2.14 Other procedures

The protein concentration was determined as reported by Bradford (1976), using bovine serum albumin as protein standard. SDS-PAGE was carried out on 1.5-mm thick slab gels containing 10 or 15% (w/v) polyacrylamide as described by Laemmli (1970). Coomassie blue R-250 or silver staining was used for protein visualization.

## 3 Results

### 3.1 Virulence of the seven Venezuelan animal trypanosome isolates

The seven trypanosome isolates showed significant differences in virulence based on the measurement of parasitaemia in infected rats. For TEVA1 and TeGu-N/D1 isolates, the parasitaemia reached ∼10^9^ trypanosomes/ml within 3–5 days, and infected animals died when the blood was not extracted in time. For the TeAp-Mantecal01 and TeAp-El Frio 01 isolates, the parasitaemia reached about 10^7^–10^9^ trypanosomes/ml over a period of 8–12 days. Finally, the TeGu-Terecay323, TeGu-Terecay03 and TeGu-TerecayTrino isolates, isolated from naturally infected donkeys, required weeks to months (5–10 weeks) to reach approximately 10^7^ trypanosomes/ml. Based on these observations, TeGu-Terecay323, TeGu-Terecay03, and TeGu-TerecayTrino were the less virulent trypanosome isolates, the virulence of TeAp-Mantecal01 and TeAp-El Frio 01 was intermediate, while TEVA1 and TeGu-N/D1 were the most virulent isolates.

### 3.2 Purification and characterization of the soluble VSG forms from seven Venezuelan animal trypanosome isolates

As shown previously for the TEVA1 isolate (Uzcanga et al., 2002, Uzcanga et al., 2004), the soluble VSG form purified for each trypanosome isolate was enriched in the flow through fraction, following chromatography of the soluble antigenic extracts on a Q-Sepharose column connected in tandem with an S-Sepharose column. In all cases, a prominent protein peak was obtained in the flow-through fraction (data not shown).

**Fig. 1:**
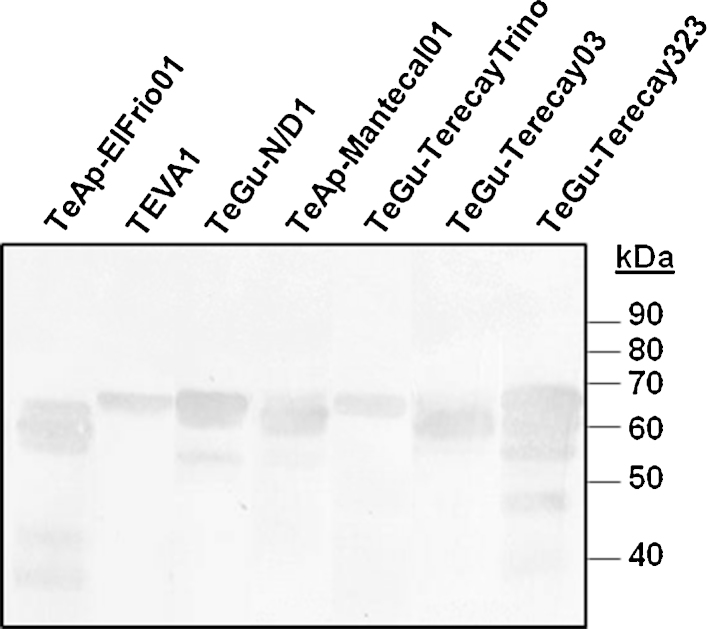
SDS-PAGE analysis of purified VSG soluble forms from Venezuelan *Trypanosoma* sp. isolates. The clarified soluble fraction from seven Venezuelan animal trypanosome isolates was chromatographed on a Q-Sepharose column connected in tandem with a S-Sepharose column. The flow-through fractions were separated by SDS-PAGE and visualized by silver staining.

SDS-PAGE analysis of the resulting protein peaks was performed under reducing conditions (Fig. 1). Major polypeptide bands with molecular masses of 48–67 kDa were observed in all samples, which may represent the soluble forms of their VSGs. The estimated sizes of the soluble VSG polypeptide bands for each trypanosome isolate were as follows: 59, 62 and 63 kDa (TeAp-El Frio01); 64 kDa (TEVA1); 56, 63 and 65 kDa (TeGu-N/D1); 62, 63 and 64 kDa (TeAp-Mantecal01); 66 kDa (TeGu-TerecayTrino); 63 kDa (TeGu-Terecay03 isolate); 48, 56, 60, 63 and 67 kDa (TeGu-Terecay323). In addition, minor polypeptide bands of lower apparent molecular weights (<48,000) were observed in some of the fractions, which likely correspond to VSG degradation products. The final yields of the purified soluble VSGs forms for each of the seven isolates were determined and corresponded to a major component of each trypanosome isolate, representing approximately 15–20% of the total parasite soluble protein.

In view of the fact that the parasite populations that were used for VSG preparation were not cloned, it is possible that the parasites harvested from a rat might contain a mixture of different VSG variants. However, since the parasites were collected on the peak of parasitaemia, and based on the fluctuating behavior of the trypanosome infection in which a clone prevails over the other clones, we expected to find a predominant VSG in infected rats and consequently, in the purified VSG fraction. If there were more than single VSG proteins in the purified fractions, they probably would correspond to minor components.

**Fig. 2:**
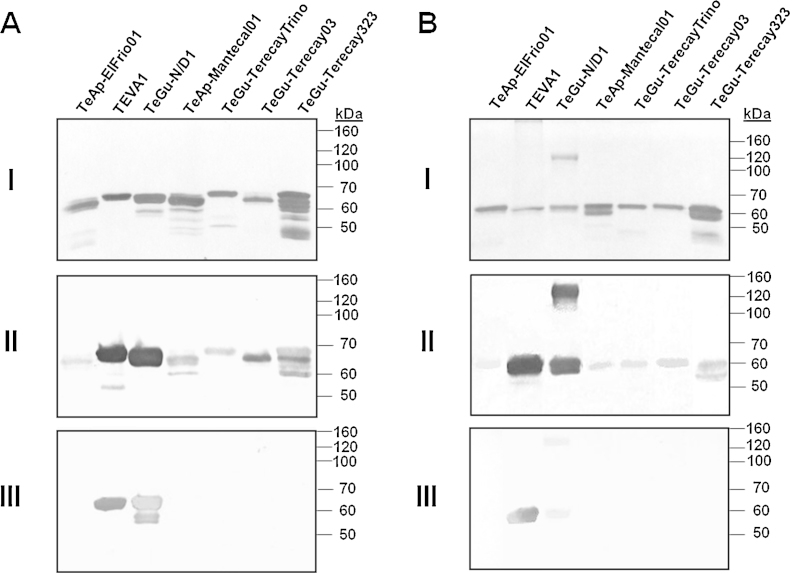
The trypanosome purified proteins are glycoproteins recognized by anti-VSG polyclonal antibodies. SDS-PAGE analysis of all purified proteins was performed under reducing (A) or non-reducing conditions (B). Proteins (5 μg) were transferred to nitrocellulose membranes and analyzed using Con A (Panel I), or polyclonal antibodies against the VSG soluble forms from the TEVA1 isolate prepared in either rabbit sera (Panel II) or mice ascitic fluid (Panel III).

To determine the glycosylation status of the VSG polypeptides, all purified proteins were separated by SDS-PAGE, transferred to a nitrocellulose membrane and analyzed by lectin blotting using Con A. The major polypeptides bands isolated from the various animal trypanosomes were recognized by Con A (Fig. 2A, Panel I). The pattern of Con A recognition resembled that of the polypeptides stained with Coomassie blue (Fig. 1). Con A binding was blocked when the incubation was undertaken in the presence of 0.2 M or 0.5 M methyl-α-d-mannopyranoside, methyl-α-d-glucopyranoside, or a mixture of both carbohydrates (data not shown). Thus, the purified proteins from the seven trypanosome isolates are glycoproteins that likely contain paucimannose or high-mannose *N*-glycans, as described for VSGs expressed by other salivarian trypanosomes (Mehlert et al., 2002). In addition, the purified soluble VSGs appeared to contain either α-d-glucose or derivatives of α-d-glucose. In the absence of β-mercaptoethanol, the glycopolypeptide bands of all samples migrated with an apparent molecular weight of approximately 60–63 kDa (Fig. 2B, Panel I). This suggests the formation of various intrachain disulfide bridges in the purified soluble form of all VSGs. Furthermore, two polypeptides were observed in the non-reducing sample of the soluble VSG forms of the TeGu-N/D1 isolate: a 60 kDa band and a 130 kDa band (Fig. 2B, Panel I). This result indicated the formation of disulfide bonds between corresponding monomers of the soluble VSG form. In contrast, there was no difference in the protein profile when the TEVA1 sample was run under non-reducing conditions. This indicates that the VSGs purified from the TEVA1 and TeGu-N/D1 isolates are products of two different genes.

Rabbit polyclonal antibodies directed against the VSG soluble form of the TEVA1 isolate were tested against the soluble VSG forms obtained from the seven different parasite isolates. These polyclonal antibodies recognized all of the soluble VSG forms, especially the proteins purified from the TEVA1 and TeGu-N/D1 isolates (Fig. 2A, Panel II). Under non-reducing conditions, these antibodies recognized the soluble VSG forms of all seven isolates (Fig. 2B, Panel II), but with lower intensity compared to the reduced samples (Fig. 2A, Panel II). Thus all the purified soluble VSG forms contained common epitopes. Moreover, the soluble VSGs from the TEVA1 and TeGu-N/D1 isolates had high similarity to each other. The same polyclonal antisera also recognized the 60 and 130 kDa polypeptide bands of the non-reducing sample of the soluble VSG forms purified from the TeGu-N/D1 isolate (Fig. 2B, Panel II). Interestingly, polyclonal antibodies produced in mice ascitic fluid against the soluble VSG forms of the TEVA1 isolate recognized only the soluble VSG forms of the TEVA1 and the TeGu-N/D1 isolates under both reducing (Fig. 2A, Panel III) and non-reducing conditions (Fig. 2B, Panel III). Together, these results demonstrated that soluble forms of different VSG variants were purified.

### 3.3 Mass spectrometry analysis of soluble VSG polypeptides

In order to corroborate the identity of the different soluble VSG preparations, samples were trypsinized in solution and the peptides were analyzed by LC/ESI-MS/MS. Analysis of the tryptic peptides from the soluble VSG forms of the TEVA1 isolate retrieved the following peptides sequences: (i) Lys-Gly-Asp-Lys-Leu-Val-Thr-Asn-Ile-Leu-Arg-Asn (KGDKLVTNILRN); (ii) Lys-Glu-Ile-Phe-Asp-Thr-Pro-Leu-Asp-Ser-Arg-Gln (KEIFDTPLDSRQ); and (iii) Lys-Ala-Leu-Thr-Ala-Leu-Ala-Thr-Ala-Ser-Glu-Arg-Asn (KALTALATASERN). According to the examined databases, the obtained sequences yielded only one hit to a putative VSG from *T*. *brucei* TREU927 (Tb927.4.5460).

A similar analysis was performed for the VSG soluble forms from the *T. evansi* TeAp-Mantecal01 isolate. The following peptides were sequenced: (i) Lys-Leu-Phe-Ala-Ala-Ile-Ala-Asn-Ala-Pro-Lys-Val (KLFAAIANAPKV); (ii) Arg-Glu-Asp-Ile-Phe-Thr-Ala-Glu-Leu-Ala-Lys-Val (REDIFTAELAKV); (iii) Arg-Ala-Val-Ser-His-Leu-Glu-Ser-Thr-Asp-Ile-Ile-Lys-Gly (RAVSHLESTDIIKG); (iv) Lys-Gly-His-Ile-Asp-Glu-Phe-Leu-Asn-Val-Ala-Glu-Lys-Val (KGHIDEFLNVAEKV); and (v) Lys-Val-Val-Asp-Ala-Thr-Phe-Ala-Asn-Ile-His-Asn-Ala-Lys-Leu (KVVDATFANIHNAKL), which matched the primary sequence of a VSG of the *T. evansi* clone ShTat1.3 (AAL15903.1). In addition, other peptide sequences were acquired during the analysis of the soluble VSG forms from the TeAp-Mantecal01 isolate. The peptide with the following sequence: Arg-Val-Ile-Leu-Pro-Ala-Val-Ala-Tyr-Gly-Gly-Glu-Val-Ala-Gly-Ala-Ile-Ser-Ser-Ala-Leu-Lys-Phe (RVILPAVAYGGEVAGAISSALKF) corresponded to another VSG from *T. evansi* (AAK49461.1). Moreover, a variety of the obtained sequences matched putative VSGs from *T. brucei*, such as: (i) Lys-Glu-Ala-Val-Val-Ala-Leu-Val-Gly-Glu-Gly-Lys-Thr (KEAVVALVGEGKT) that hit a VSG from *T. brucei* TREU927 (Tb927.6.5450); (ii) Arg-Ala-Ala-Ile-Tyr-Ser-Gln-Leu-Gln-Lys-Gly (RAAIYSQLQKG) that hit another VSG from *T. brucei* TREU927 (Tb927.5.230); and (iii) Lys-Leu-Ile-Thr-His-Val-His-Val-Glu-Ala-Arg-Cys (KLITHVHVEARC) that also hit a putative VSG from *T. brucei* (Q4FKD2 or Tb09.244.1350). Although no sequence information was attained for the rest of the purified proteins, the results obtained for the TEVA1 and TeAp-Mantecal01 proteins indicated that the purified proteins corresponded to the soluble form of VSG variants from animal trypanosomes.

### 3.4 Gel filtration chromatography

**Fig. 3:**
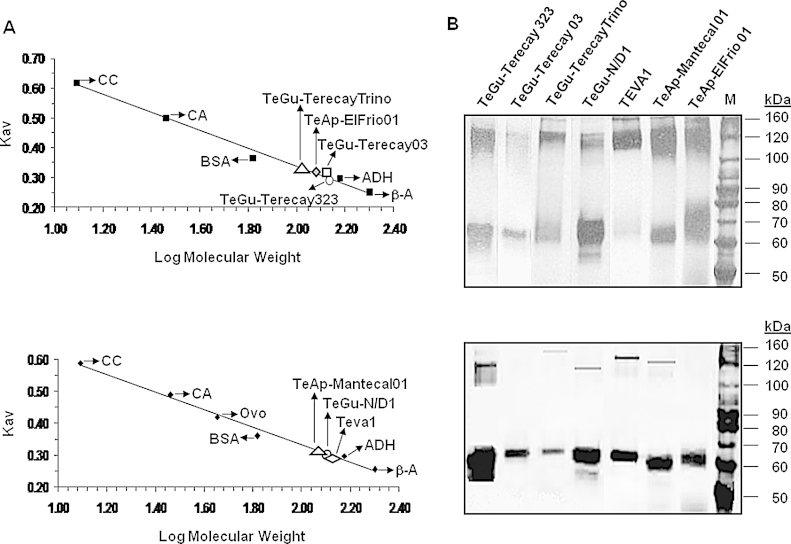
Gel filtration chromatography and cross-linking of the purified soluble VSGs. Panel A: The native molecular weight of the purified proteins was estimated using a Sephacryl S-300 molecular exclusion column. The calibration curve was established with β-amylase (β-A, 200 kDa), alcohol dehydrogenase (ADH, 150 kDa), bovine serum albumin (BSA, 67 kDa), ovoalbumin (Ovo, 43 kDa), carbonic anhydrase (CA, 29 kDa), and cytochrome C (CC, 12.4 kDa). Elution positions of the purified VSG soluble forms were determined by SDS-PAGE and immunoblotting using sera from a bovine experimentally infected with *T. vivax*. Panel B: Samples of the purified soluble form of each VSG (8 μg) were incubated with 5 mM sulfo-SMCC (top panel) or *o*-PDM (bottom panel), for 1 h at room temperature. M, protein markers.

Samples of all purified VSG soluble forms were applied to a Sephacryl S-300 molecular exclusion column with a set of protein markers. SDS-PAGE and Western blot analyses, using sera from an experimentally infected cow, allowed estimation of the elution volume of each purified protein. The purified protein sizes were empirically determined by plotting the *K*_*av*_ value of each standard versus the logarithm of its molecular weight (Fig. 3A). Molecular masses of 130 kDa, 140 kDa, 138 kDa, 134 kDa, 122 kDa, 137 kDa, and 139 kDa for the soluble VSG forms purified from TeAp-El Frio01, TEVA1, TeGu-N/D1, TeAp-Mantecal01, TeGu-TerecayTrino TeGu-Terecay03, and TeGu-Terecay323, respectively, were estimated, demonstrating that all purified proteins correspond to dimers in their native form. A dimeric quaternary structure is a common attribute of reported VSGs from salivarian trypanosomes.

### 3.5 Covalent cross-linking of the purified VSG soluble forms

Samples of all purified VSG soluble forms were incubated with sulfo-SMCC, *o*-PDM or *p*-PDM; three bifunctional reagents capable of covalently linking proteins. Sulfo-SMCC is a heterobifunctional cross-linker capable of forming bridges between Cys and Lys spatially located about 11.6 Ǻ apart. *o*-PDM and *p*-PDM are specific homobifunctional agents that cross-link Cys residues located at approximately 9 Ǻ and 12 Ǻ, respectively. Incubation of the purified soluble VSG forms with sulfo-SMCC produced diffuse polypeptides bands that migrated at approximately 118–140 kDa (Fig. 3B, top panel). Although *p*-PDM did not cross-link any of the purified soluble VSGs (data not shown), *o*-PDM was capable of cross-linking some of the VSG variant soluble forms (Fig. 3B, bottom panel) and produced polypeptides bands that migrated at approximately 118–160 kDa. The molecular weights obtained suggested the formation of dimeric cross-linked products, supporting the fact that purified soluble VSGs are dimers. As expected, the migration of all purified proteins by SDS-PAGE was not modified in the control samples (data not shown).

### 3.6 Isoelectric point determination

**Fig. 4:**
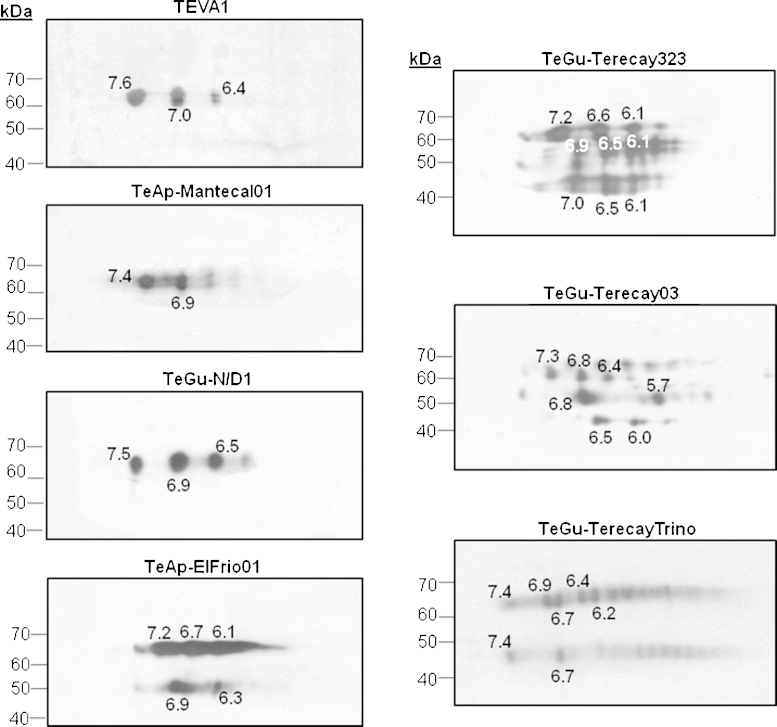
Two-dimensional electrophoresis of the purified soluble VSGs. IEF was performed using polyacrylamide strips containing a linear ampholyte gradient (pH 5–8). Following the initial fractionation, strips containing the separated proteins were incubated with an appropriate concentration of SDS and placed on top of a slab gel containing 10% (w/v) polyacrylamide. The second dimension was performed as described by Laemmli (1970) and the gels were silver stained. The isoelectric points of the various VSG isoforms found for each of the seven animal trypanosome isolates are shown.

Two-dimensional electrophoresis of the VSG soluble forms purified from all seven isolates of *Trypanosoma* sp. was performed (Fig. 4). Using protein markers, the isoelectric points and molecular masses of all soluble VSG variants were calculated. As expected, all the soluble VSGs gave p*I* values between 6.1 and 7.5 and there were at least three isoforms of each of the purified proteins. The results obtained were as follows: (i) TEVA1: three spots with an apparent molecular mass of 64 kDa and p*I* values of 6.4, 7.0 and 7.6; (ii) TeAp-Mantecal01: two spots with an apparent molecular mass of 62 kDa and p*I* values of 6.9 and 7.4; (iii) TeGu-N/D1: three spots with an apparent molecular mass of 63 kDa and p*I* values of 6.5, 6.9 and 7.5; (iv) TeAp-ElFrio01: five spots, three with an apparent molecular mass of 62 kDa and p*I* values of 6.1, 6.7 and 7.2, and two spots with an apparent molecular mass of 45 kDa and p*I* values of 6.3 and 6.9; (v) TeGu-Terecay 323: nine spots, three with an apparent molecular mass of 67 kDa and p*I* values of 6.1, 6.6 and 7.2, three with apparent molecular masses of 61–63 kDa and p*I* values of 6.1, 6.5 and 6.9, and three with an apparent molecular mass of 58 kDa and p*I* values of 6.1, 6.5, and 7.0; (vi) TeGu-Terecay03: three spots with an apparent molecular mass of 63 kDa and p*I* values of 6.4, 6.8, and 7.3; and (vi) TeGu-TerecayTrino: seven spots, five with an apparent molecular mass of 66 kDa and p*I* values of 6.2, 6.4, 6.7, 6.9 and 7.4, and two with an apparent molecular mass of 45 kDa and p*I* values of 6.7 and 7.4. Spots of lower apparent molecular weights (<58,000) were observed in TeAp-ElFrio01, which required approximately 12 days to reach the appropriate parasitaemia; and in TeGu-Terecay323, TeGu-Terecay03, and TeGu-TerecayTrino, which were the less virulent trypanosome isolates and required 5–10 weeks to reach the proper parasitaemia. These spots likely correspond to VSG degradation products, which may be accounted for by the variation in parasitaemia observed between these different trypanosome isolates.

### 3.7 The soluble VSG forms purified from seven different Venezuelan trypanosome isolates are recognized by sera from animals naturally infected with either *T. evansi* or *T. vivax*

**Fig. 5:**
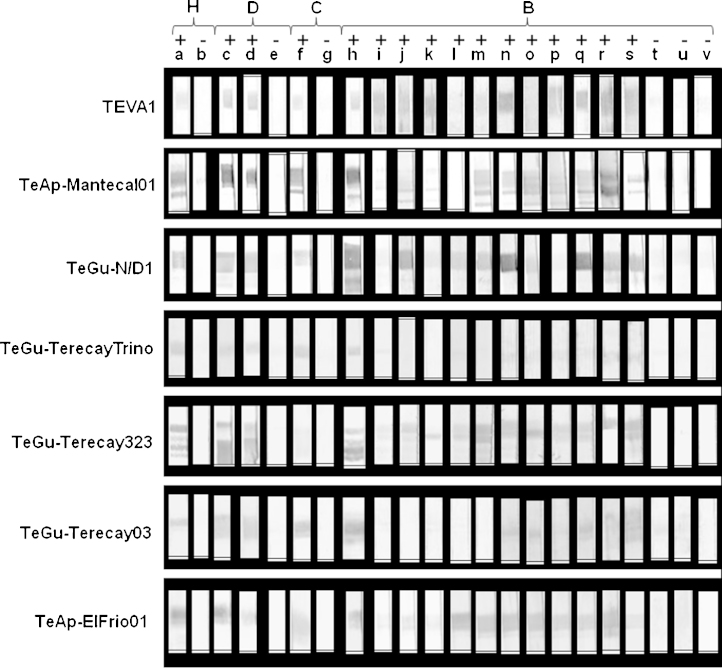
Purified soluble VSGs are recognized by sera from *T. evansi*- and *T. vivax*-infected animals. Western blotting was performed using sera from naturally infected horses (H), donkeys (D), capybaras (C), or bovines (B) to evaluate recognition of the purified proteins. Sera from experimentally infected animals were included as positive controls. Sera from uninfected (−) and infected animals (+) were used.

Western blot analysis was undertaken to evaluate recognition of the purified proteins by sera from horses, donkeys, capybaras and cows naturally infected with either *T. evansi* or *T. vivax*. Sera from experimentally infected animals were included as positive controls. The purified soluble VSG forms were recognized by sera obtained from a horse and a cow experimentally infected with *T. evansi* and *T. vivax*, respectively (Fig. 5, Lanes a and h), but not by serum from an uninfected racehorse (Fig. 5, Lane b). The purified proteins from all *T. evansi* isolates were also recognized by sera obtained from donkeys (Fig. 5, Lanes c and d) and a capybara (Fig. 5, Lane f) that had been naturally infected with *T. evansi*, but not by sera from an uninfected donkey (Fig. 5, Lane e) or an uninfected capybara (Fig. 5, Lane g). Furthermore, purified proteins were not recognized by sera from uninfected cows (Fig. 5, Lanes t, u, and v). Interestingly, most of the purified soluble VSG forms from different trypanosome isolates were well recognized by sera from cows naturally infected with *T. vivax* (Fig. 5, Lanes i–s). However, soluble VSG forms from the TeGu-TerecayTrino and TeGu-Terecay03 isolates were weakly recognized by sera from cows naturally infected with *T. vivax* (Fig. 5, Lanes i–s).

### 3.8 The inositol-1,2-cyclic phosphate moiety of the CRD is partially responsible for the cross-reactivity of the purified VSG soluble forms

**Fig. 6:**
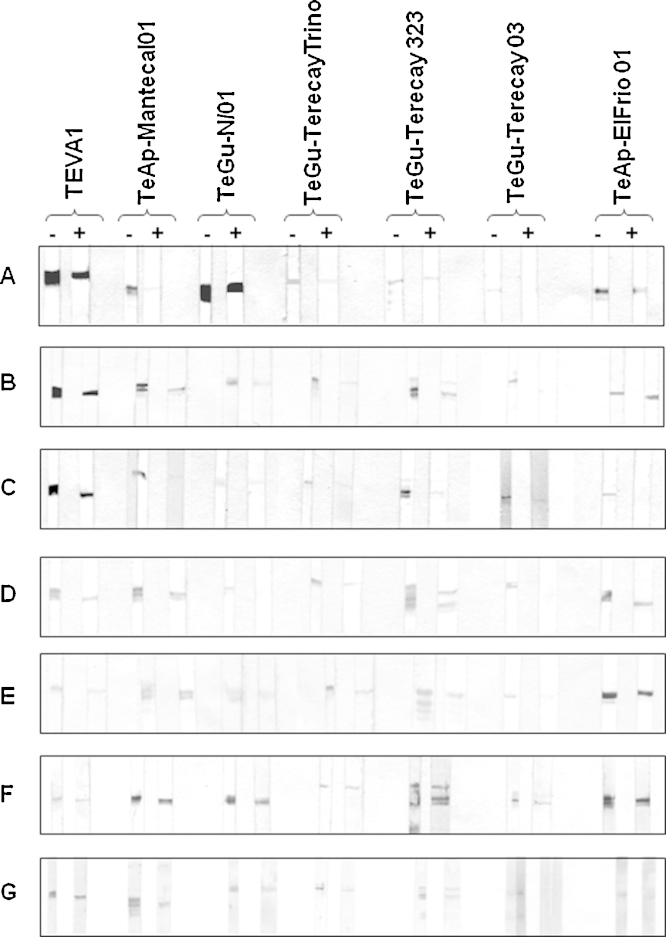
Effect of mild acid treatment on the cross-reaction of the purified proteins. The soluble VSG forms of the seven Venezuelan isolates of *Trypanosoma sp.* were incubated either with 1 M HCl (+) or without (−), for 1 h, at room temperature. Samples were separated by SDS-PAGE, and transferred to nitrocellulose sheets. The blots were cut into 4 mm strips and developed using either rabbit polyclonal antibodies directed to the soluble VSG form of the TEVA1 isolate (Panel A) or sera from animals either naturally or experimentally infected with trypanosomes (Panel B–G). Sera from a bovine (B), a buffalo (C), a capybara (D), a horse (E) and two donkeys (F and G) naturally infected with trypanosomes were used.

Rabbit polyclonal antibodies against the soluble VSG forms of the TEVA1 isolate (Fig. 6A) and sera from a cow (Fig. 6B), a buffalo (Fig. 6C), a capybara (Fig. 6D), a horse (Fig. 6E) and two donkeys (Fig. 6F and G), which had been naturally infected with trypanosomes, were used to monitor the CRD-dependent cross-reactivity of the purified proteins from the seven Venezuelan trypanosome isolates. Samples of all soluble VSG forms were treated (+) with 1 M HCl or left untreated (−) (Schneider et al., 1990). Mild acid treatment causes decyclization of the inositol-1,2-cyclic phosphate moiety of the CRD in vitro (Zamze et al., 1988, Schneider et al., 1990) and destroys the antigenicity of the inositol structure.

As shown previously (Fig. 2A, Panel II), rabbit polyclonal antibodies against the soluble VSG forms of the TEVA1 isolate recognized all seven soluble untreated VSG forms, especially proteins purified from the TEVA1 and TeGu-N/D1 isolates (Fig. 6A, −). All purified proteins were recognized by sera obtained from a cow (Fig. 6B, −) and a horse (Fig. 6E, −) that had been experimentally infected with *T. vivax* and *T. evansi*, respectively. Also, they were recognized by sera obtained from a buffalo (Fig. 6C, −), a capybara (Fig. 6D, −) and two donkeys (Fig. 6F, − and G, −) that had been naturally infected with trypanosomes. These results confirmed that the purified soluble VSG forms from the various trypanosome isolates were antigens partially responsible for the cross-reactivity between *T*. *evansi* and *T*. *vivax*.

In most cases, there was only a slight decrease in the recognition of the seven antigens by all sera following mild acid treatment (Fig. 6A–G, +). These results indicated that the inositol-1,2-cyclic phosphate moiety of the CRD is only partially responsible for the cross-reactivity of the purified proteins. However, a complete blockage of recognition by several animal sera was observed following mild acid treatment of the soluble VSG form of the TeGu-Terecay03 isolate (Fig. 6).

### 3.9 Limited proteolysis with *S. aureus* V8 protease of the seven purified soluble VSGs

**Fig. 7:**
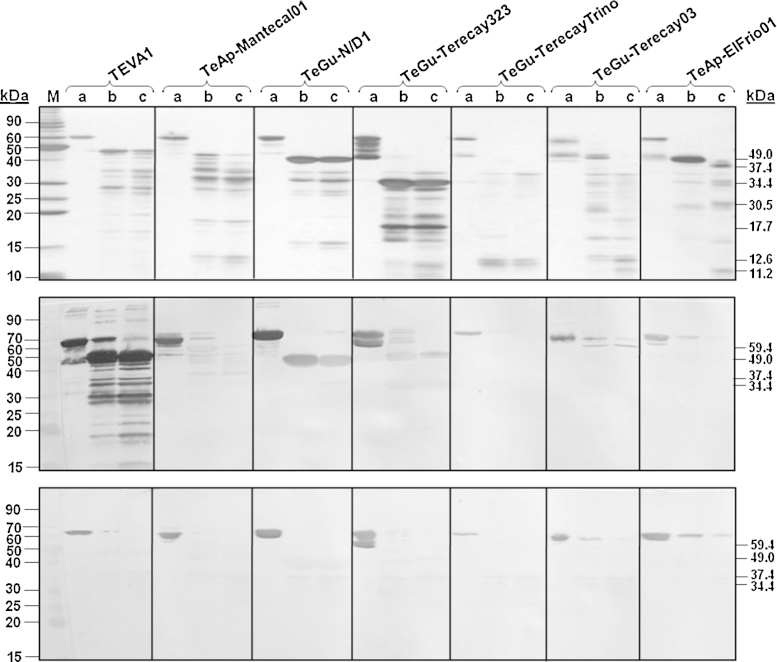
Limited proteolysis of the soluble VSGs using *S. aureus* V8 protease. Samples of each purified protein (40 μg) were either left untreated (a), or partially digested with 1.12 (b) or 0.37 (c) units of *S. aureus* V8 protease for 4.5 h at room temperature. SDS-PAGE analysis of these samples was performed using a 15% (w/v) polyacrylamide gel. Gels were either silver stained (top panel) or analyzed by Western blotting using polyclonal antibodies produced in rabbit against the soluble VSG form from TEVA1 (middle panel), or serum from a bovine naturally infected with *T. vivax* (bottom panel).

Following limited proteolysis with *S. aureus* V8 protease, the resulting polypeptide fragments were compared by SDS-PAGE analysis to examine similarities among the primary structures of the seven purified proteins. Common proteolytic profiles are expected for protein variants that possess conserved three-dimensional folding. Although some clear differences were evident, the generated V8 proteolytic patterns for the seven soluble VSGs were similar, showing polypeptide bands with apparent molecular masses of 49, 37.4, 34.4, 30.5, 17.7, 12.6 and 11.2 kDa (Fig. 7, top panel). As expected, higher digestion of the purified proteins occurred when more (Fig. 7, top panel, Lane c) rather than less (Fig. 7, top panel, Lane b) *S. aureus* V8 protease was used.

To identify antigenic regions within the purified soluble VSGs, the proteolytic fragments were analyzed by immunostaining. Polyclonal antibodies produced in rabbits against the soluble VSG forms of the TEVA1 isolate (Fig. 7, middle panel) and serum from a bovine naturally infected with *T. vivax* (Fig. 7, bottom panel) were used. Both sera recognized all the untreated soluble VSGs, and some of their proteolytic fragments. However, recognition of the soluble VSG forms of TEVA1, as well as its proteolytic fragments, was higher when the polyclonal antibodies were used. In particular, a major polypeptide fragment of about 49 kDa was attained when the purified VSG from TEVA1 was treated with the V8 protease. These antibodies also recognized polypeptide bands between 18 and 40 kDa in size of the TEVA1 VSG variant (Fig. 7, middle panel). Low recognition of the resulting proteolytic fragments of the soluble VSGs of the other trypanosome isolates was observed when these antibodies were employed, with only some recognition in the 40–60 kDa region (Fig. 7, middle panel). Lower recognition of the proteolytic fragments was attained when the bovine serum was used (Fig. 7, bottom panel). Common proteolytic bands, with molecular masses of approximately 59.4, 49, 37.4, 34.4 and 33 kDa (Fig. 7, bottom panel) were barely detected in most purified soluble VSGs. This suggests that antibodies against the soluble VSG forms of the TEVA1 isolate and antibodies in the serum of the bovine naturally infected with *T. vivax* recognized cross-reacting conformational epitopes from the various soluble VSGs, which were lost following proteolysis with the V8 protease.

### 3.10 Recognition of the purified soluble VSG forms by sera from bovine living in a trypanosomosis-endemic area

**Fig. 8:**
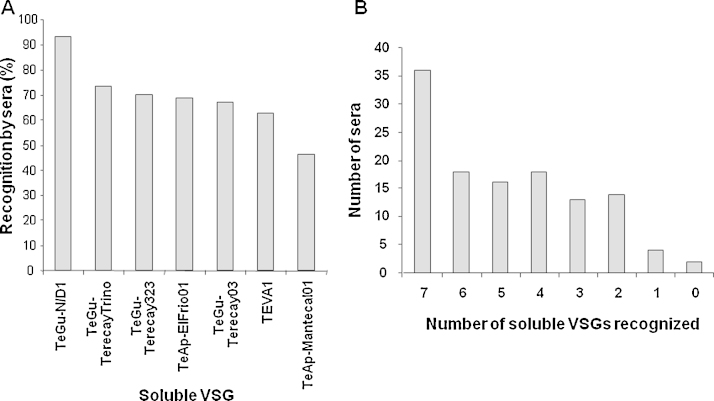
Indirect ELISA of the soluble VSGs using bovine sera obtained from a trypanosomosis-endemic region of Venezuela. ELISA plates were sensitized with the purified soluble VSG forms of the seven different trypanosome isolates (1 μg of protein/well) and 121 bovine sera (dilution 1:100) obtained from a trypanosomosis-endemic area of Venezuela were evaluated. Panel A: % bovine sera samples that recognized each of the purified soluble VSG forms from the seven Venezuelan *Trypanosoma* sp. isolates. Panel B: the number of sera that recognized 1–7 of the purified VSG soluble forms.

Indirect ELISA was used to evaluate recognition of the purified soluble VSG forms from different trypanosome isolates by sera from bovine living in a trypanosomosis-endemic region of Guárico State, Venezuela (Fig. 8A). Most purified soluble VSGs were recognized by >60% of the sera. However, soluble VSG from the TeAp-Mantecal01 isolate was recognized by only 46% of the bovine sera. Notably, the soluble VSG from the TeGu-N/D1 isolate was recognized by 93.38% of the bovine sera samples tested. Soluble VSG from the TeGu-TeracayTrino isolate was recognized by 73.55% of the sera. Thirty-six sera (corresponding to 29.8% of the total sera samples tested) recognized the purified soluble VSGs obtained from the seven trypanosome isolates. Only four sera recognized a single soluble VSG sample, which corresponded to the variant from the TeGu-N/D1 isolate (Fig. 8B).

## 4 Discussion

The diagnostic value of VSG molecules in *T. evansi* and *T*. *brucei* infections has been extensively reported (Van Meirvenne et al., 1977, Magnus et al., 1982, Van Meirvenne et al., 1995, Verloo et al., 2000, Holland et al., 2002, Atarhouch et al., 2003, Rogé et al., 2013). Currently, native and recombinant VSG antigens are used in most antibody detection and DNA-based tests for salivarian trypanosomes. For example, an indirect latex agglutination test (LATEX/*T.b.g*), consisting of a lyophilized suspension of latex particles coated with VSGs of the variable antigen types LiTat 1.3, LiTat 1.5 and LiTat 1.6 from *T. brucei gambiense*, has been successfully employed for diagnosis of human African trypanosomosis (Penchenier et al., 2003). Most *T. evansi* populations can also be identified using diagnostic assays. These include direct card agglutination tests (CATT/*T. evansi*) (Bajyana Songa and Hamers, 1988, Verloo et al., 2000, Atarhouch et al., 2003, Holland et al., 2002), indirect latex agglutination tests (LATEX/*T. evansi*) (Verloo et al., 2000, Holland et al., 2002) or PCR assays (Ngaira et al., 2004), and are based on detection of the predominant *VSG* gene of *T. evansi* Rode Trypanozoon antigen type (RoTat) 1.2, a variable antigen type that has been cloned from a *T. evansi* stock, isolated in 1982 from a water buffalo in Indonesia (Bajyana Songa and Hamers, 1988). These tests have shown high specificity and sensitivity. However, the RoTat 1.2 *VSG* gene is absent in some *T. evansi* trypanosomes (Ngaira et al., 2004, Salim et al., 2011). Therefore, use of tests based only on the RoTat 1.2 *VSG* gene can potentially lead to false-negative results. Thus, novel diagnostic methods for the detection of animal trypanosomosis are needed.

Various recent studies have described several promising recombinant proteins for the serodiagnosis of *T. evansi* and *T. vivax*. Tran et al. (2009) generated an antibody detection ELISA for *T. evansi* infection in camels by using a recombinant extracellular domain of an invariant surface glycoprotein 75 (rISG75) expressed in *Escherichia coli* (ELISA/rISG75). The ELISA/rISG75 demonstrated an almost perfect agreement with three established antibody detection tests based on VSG RoTat 1.2: an ELISA for *T. evansi*, the CATT/*T. evansi* card agglutination test for trypanosomosis, and an immune trypanolysis assay. Moreover, ELISA/rISG75 tests on a small serum sample set of *T. brucei brucei*-infected goats, *T. evansi* RoTat 1.2-infected horses, and *T. equiperdum*-infected rabbits showed that rISG75 was panreactive within the Trypanozoon subgenus. Since tandem repeat proteins of trypanosomatid parasites generally possess high antigenicity, they also have been considered to be potential antigens for trypanosomosis. The repeat sequences of the flagellar-associated GM6 proteins of *T. vivax* (TvGM6) and *Trypanosoma* (Nannomonas) *congolense* (TcoGM6) were recombinantly expressed and purified (Pillay et al., 2013). The purified GM6 antigens were subsequently used in an indirect ELISA that was optimized for detection of trypanosome infection in bovine sera. Pillay et al. (2013) demonstrated that the TvGM6 was an excellent candidate antigen for the development of a point-of-treatment test for diagnosis of *T. vivax*, and to a lesser extent *T. congolense*, African animal trypanosomosis in cattle. In addition, recombinant *T. b. brucei* GM6 (TbbGM6) and *T. evansi* GM6 (TeGM6-4r) were proven to be valuable as serodiagnostic antigens for *T. evansi* infection (Thuy et al., 2012, Nguyen et al., 2014). Although the use of recombinant antigenic proteins of *T. evansi* and *T. vivax* may be an alternative source of antigens which appeared to be very useful for the serodiagnosis of animal trypanosomosis, given that they can be highly reproducible and have the additional benefit that laboratory animal usage is reduced, native antigens have the advantage that they possess the original three dimensional conformation of the protein and bear all co- and post-translational covalent modifications, which might represent essential epitopes for antigen immunoreactivity. As shown here, not only the protein component but also the inositol-1,2-cyclic phosphate moiety of the glycosyl-phosphatidylinositol anchor is partially responsible for the cross-reactivity of the purified VSG soluble forms. In addition, unpublished results using oxidation with sodium metaperiodate followed by sodium borohydride reduction have shown that carbohydrate epitopes are also important for the immunorecognition of the purified VSG soluble antigens (Uzcanga G.L., unpublished results). Recently, Fikru et al. (2014) also developed a *T. vivax* specific PCR test based on the *T. vivax* proline racemase (*TvPRAC*) gene, which proved to be fully specific for *T. vivax*, irrespective of its geographical origin.

In this study, we purified the soluble forms of the predominant VSG variants from seven Venezuelan isolates of animal trypanosomes originally obtained from naturally infected horses, donkeys and a capybara. The biochemical and immunological characterization of the purified proteins revealed that each of these variants corresponded to different VSG molecules. The purified soluble VSGs of the TEVA1 and TeAp-Mantecal01 trypanosome isolates were analyzed by liquid chromatography-electrospray tandem mass spectrometry. Three of the tryptic peptides sequenced from the purified soluble VSG of the TEVA1 isolate generated only one hit with a *T. brucei* TREU927 gene that encoded for a putative VSG (Tb927.4.5460). However, the theoretical isoelectric point for the Tb927.4.5460 gene product, determined using the Compute pI/Mw tool (http://web.expasy.org/compute_pi/) was 8.69, which differed from the experimentally obtained values for the isolelectric point of the three isoforms isolated from TEVA1. In addition, the rest of the tryptic peptides that were sequenced from the soluble VSG forms of the TEVA1 isolate did not match any of the VSGs reported in the kinetoplastid genomics resource database (TriTrypDB, http://tritrypdb.org/tritrypdb/). Therefore, VSG isoforms from TEVA1 are probably products of gene recombination in which the homologous gene for Tb927.4.5460 is involved. Given that VSGs may enter a telomeric expression site via homologous recombination, there is potential for the formation of new VSGs that are mosaics of existing VSGs (Marcello and Barry, 2007). Five peptides from the soluble VSGs of the TeAp-Mantecal01 *T. evansi* isolate matched with the amino acid sequence of the AAL15903.1 VSG gene product from the *T. evansi* clone ShTat1.3, and another peptide matched with the *T. evansi* AAK49461.1 VSG gene product. VSGs AAL15903.1 and AAK49461.1 have theoretical isoelectric points of 7.51 and 6.59, respectively, which are similar to the experimental isoelectric points (7.4 and 6.9) obtained for the soluble VSG forms from the TeAp-Mantecal01 *T. evansi* isolate. In addition, the last 38 amino acids of the VSG AAL15903.1 gene product were identical to the C-terminal amino acid sequence from a VSG of the *T. evansi* YNB isolate (VSG YNBC2), which was originally isolated in 1987 from a naturally infected buffalo in the Yunnan province, China (Jia et al., 2011). Although VSG YNBC2 does not have any potential *N*-glycosylation sites (Jia et al., 2011), the soluble VSG forms isolated from the TeAp-Mantecal01 *T. evansi* isolate were identified as glycoproteins in this study. Therefore, the VSGs from the TeAp-Mantecal01 *T. evansi* isolate also appear to be a homologous recombination product of the VSG AAL15903.1, the VSG AAK49461.1 and the VSG YNBC2 genes.

Rabbit polyclonal antibodies directed against the VSG soluble form of the TEVA1 isolate were capable of recognizing all of the soluble VSG forms. The rabbit polyclonal antibodies were capable of recognizing either the CRD, an antigenic component common to all VSG soluble forms, or some conformational epitopes of the various VSG variants. In contrast, polyclonal antibodies produced in mice ascitic fluid against the soluble VSG forms of the TEVA1 isolate recognized only the soluble VSG forms of the TEVA1 and the TeGu-N/D1 isolates. Thus, the polyclonal antibodies produced in mice ascitic fluid did not recognize the CRD component of all soluble VSG forms of the different trypanosome isolates. In *T. brucei*, the *S. aureus* V8 protease cleaves some soluble VSG variants into an N-terminal domain and a C-terminal domain containing 350–400 residues and 50–100 residues, respectively (Freymann et al., 1984). Similarly, the purified soluble VSGs presented in this study were digested by the *S. aureus* V8 protease into N-terminal and C-terminal fragments. The proteolytic fragments obtained after digestion of the soluble VSGs of the TEVA1 and TeGu-N/D1 isolates with the *S. aureus* V8 protease were also recognized by the polyclonal antibodies produced in mice ascitic fluid against the soluble VSG forms of the TEVA1 isolate. However, the *S. aureus* V8 protease-digested fragments of the other five purified soluble VSGs were not well-recognized when these antibodies were employed. Thus, the soluble VSGs from the TEVA1 and TeGu-N/D1 isolates appeared to be more similar to each other than to the purified proteins of the other isolates. Our results also showed low recognition of the individual V8 proteolytic fragments by sera from bovines naturally infected with *T. vivax*. Thus the cross-reacting epitopes that were recognized in the purified soluble VSGs appeared not to be linear but structural or conformational. Reactivity of the fragments completely disappeared when the higher proteolytic fragments of the soluble VSGs, which are associated with the N-terminal domain of the proteins, were cleaved. Sengupta et al. (2012) expressed a recombinant truncated protein corresponding to the N-terminal domain of the *T. evansi* RoTat 1.2 VSG. This protein remained reactive with all combinations of sera from buffalo, dog, lion and leopard infected with *T. evansi*. Similar antibody reactions by ELISA and CATT were observed for animals immunized with either the whole parasite lysate or the truncated N-terminal portion of the RoTat 1.2 VSG. A plausible explanation is that all *T. evansi* strains that infected the animals from which the sera were tested, expressed the RoTat 1.2 VSG, as seen by Verloo et al. (2001) with a collection of *T. evansi* populations from South America, Asia and Africa that were isolated from various host species. Alternatively, another reasonable explanation is that the VSG N-terminal domain contains an important cross-reacting epitope. Van Nieuwenhove et al. (2012) searched for mimotopes with diagnostic potential for *T. brucei gambiense* VSG LiTat 1.3 and VSG LiTat 1.5. They identified a sequence in the N-terminal region of these proteins that appears to be responsible for epitope formation. More recently, Van Nieuwenhove et al. (2013) tested and compared a synthetic biotinylated peptide corresponding to amino acids 268–281 of the VSG LiTat 1.5 (peptide 1.5/268–281), and native VSGs LiTat 1.3 and LiTat 1.5 in an indirect ELISA with 102 sera from patients with human African trypanosomosis and 102 endemic human African trypanosomosis-negative controls. They concluded that the biotinylated peptide 1.5/268–281 may replace native VSGs in serodiagnostic tests. However, the diagnostic accuracy obtained was lower than that acquired for the full-length native VSG LiTat 1.3 and VSG LiTat 1.5. Similarly to the RoTat 1.2 VSG, VSG LiTat 1.3 and VSG LiTat 1.5 appear to have reactive epitopes in the hypervariable N-terminal domain of the protein.

All seven trypanosome isolates utilized in this study were previously identified as *T. evansi* using both Trypanozoon primers and specific primers for *T. evansi*. However, Perrone et al. (2009) compared these *Trypanosoma* sp. isolates using the RAPD technique. They found that TEVA1 and TeGu-N/D1 had a remarkable 96.7% similarity and were genetically polymorphic when compared to the remaining *T. evansi* isolates, with similarity coefficients of between 57.9% and 68.4%. Therefore, they proposed that the TEVA1 and TeGu-N/D1 isolates belong to a morphologically indistinguishable species within the subgenus Trypanozoon, such as *T. equiperdum*, which has not been reported in Venezuela before, and indicated that the rest of the isolates corresponded to *T. evansi*. However, Masiga et al. (2006) proposed that *T. evansi* is a polyphyletic species. By analyzing population genetics of *T. evansi* using amplified restriction fragment length polymorphism, they suggested that there were two independent origins of *T. evansi* from *T. brucei*, and classified the *T. evansi* isolates into two genetically quite different types, type A and type B. Type A spread to its current worldwide distribution, whereas type B remained more local to East Africa. Hence, the polyphyleticity of *T. evansi* may also explain the genetic heterogeneity observed by Perrone et al. (2009) when these trypanosome isolates were compared by RAPDs. These alternative interpretations have generated controversy regarding the correct definition of the species to which each of the trypanosome isolates belong. It would be interesting to characterize all seven of the Venezuelan trypanosome isolates used in this study by performing PCR analysis, using: (i) primers derived from the sequence of the maxicircle kDNA of *T. brucei* encoding the NADH dehydrogenase subunit 5 (*nad5*) gene (Li et al., 2007); (ii) *T. evansi* type B specific primers EVAB1 and EVAB2, which were designed from the minicircle sequence (Njiru et al., 2006), and (iii) primers targeting the inter-specific length variation of the internal transcribed spacer (ITS) regions of ribosomal genes, which have been reported to differ among trypanosome species (Cox et al., 2005).

The whole genome sequencing of *T. evansi* is still ongoing and data for this organism is not completely available yet (https://www.sanger.ac.uk/resources/downloads/protozoa/trypanosoma-evansi.html). However, our understanding of how antigenic diversity is organized has been greatly improved by the *T. brucei* reference genome sequence (Berriman et al., 2005). Recently, Jackson et al. (2012) compared the genome of *T. brucei* with *T. congolense* and *T. vivax*, and revealed how the variant antigen repertoire has evolved and how it might affect contemporary antigenic diversity. They reconstructed VSG diversification showing that *T. congolense* uses variant antigens derived from multiple ancestral VSG lineages, whereas in *T. brucei* VSG have recent origins and ancestral gene lineages have been repeatedly co-opted to novel functions. Using phylogenetic incompatibility as a metric for genetic exchange, Jackson et al. (2012) showed that the frequency of recombination was comparable between *T. congolense* and *T. brucei* but was much lower in *T. vivax*. Moreover, Jackson et al. (2012) also showed that VSG structural diversity was greater in *T. vivax* than in *T. brucei* and *T. congolense*. In this study, immunological analysis showed that sera from *T. vivax*-positive bovines recognize the soluble forms of the predominant VSGs expressed by seven different Venezuelan isolates of trypanosome species during the early infection stages. Some of the *T. vivax*-infected bovine sera recognized all purified soluble VSG variants, while sera from other infected animals recognized only a selection of the isolated soluble VSGs. Interestingly, despite the huge repertoire of *VSG* genes existing on salivarian trypanosome genomes and their hypervariability, our findings clearly revealed that VSGs are antigens that contain common epitopes which are recognized by sera from animals infected with either *T. evansi* or *T. vivax.* Moreover, anti-VSG antibodies appeared to behave as markers of infection for non-tsetse transmitted trypanosomes due to the cross-reactivity exhibited by their VSGs. Notably, the soluble VSG of the TeGu-N/D1 isolate was the best cross-reacting antigen of all the purified soluble variants, recognizing 93.38% of the bovine sera collected from a trypanosomosis-endemic area of Venezuela. Thus this VSG may be an important candidate for use in a diagnostic test. In spite of all of these results, only about 30% of the sera were capable of recognizing all seven purified VSGs. Therefore, we suggest the use of a combination of VSGs as diagnostic reagents for animal trypanosomosis, instead of a single VSG.

## 5 Conclusions

We purified the soluble forms of the predominant VSG variants expressed by seven Venezuelan isolates of animal trypanosomes originally obtained from naturally infected horses, donkeys and a capybara. The biochemical characterization and immunological evaluation of the purified proteins revealed that each of these variants corresponded to different VSG molecules. Interestingly, all purified VSGs showed cross-reactivity with *T. vivax*. Despite the huge repertoire of *VSG* genes on these trypanosome genomes and their variability, our results demonstrate the potential use of VSG variants, either singly or in combination, for the diagnosis of non-tsetse transmitted animal trypanosomosis.

## Conflict of interests

The authors declare that there is no conflict of interests regarding the publication of this article.

## Ethical standards

We declare that all the experiments in this paper were carried out in accordance with the legal and ethical standards of Venezuela.
